# The Characteristics of the Root-Zone Soil’s Biological Properties and Microbial Community Structure in Grafted Star Anise Plantations

**DOI:** 10.3390/microorganisms12030431

**Published:** 2024-02-20

**Authors:** Jian Xiao, Junxian Liu, Siyu Wu, Wenhui Liang, Shangdong Yang

**Affiliations:** 1 Guangxi Key Laboratory of Agro-Environment and Agro-Products Safety, National Demonstration Center for Experimental Plant Science Education, Agricultural College, Guangxi University, Nanning 530004, China; hn_xiaojian@163.com; 2 Agricultural Resources and Environmental Research Institute, Guangxi Academy of Agricultural Sciences/Guangxi Key Laboratory of Arable Land Conservation, Nanning 530007, China; 3 Longping Branch, College of Biology, Hunan University, Changsha 410125, China; 4 Guangxi Key Laboratory of Sugarcane Genetic Improvement, Guangxi Academy of Agricultural Sciences, Nanning 530007, China; liujunxian868@163.com; 5 Guangxi Key Laboratory of Special Non-Wood Forest Cultivation & Utilization, Guangxi Forestry Research Institute, Engineering and Technology Research Center for Anise and Cinnamon of State Forestry and Grassland Administration, Guangxi Engineering and Technology Research Center for Woody Spices, Nanning 530002, China; wusiyupp@163.com (S.W.); l.wenhui@163.com (W.L.)

**Keywords:** star anise, grafted, soil biological properties, soil microbial community

## Abstract

Extensive management seriously affects the output, quality, and sustainable development of star anise, and grafting is commonly used to improve its production and quality. Although many studies have explored the effects of grafting on soil microorganisms for other plants, there is a lack of research on aromatic plants, especially on the soil ecosystems of star anise plantations. The effect of grafting star anise on the soil’s biological characteristics and microbial composition remains unclear. The soil’s enzyme activities, soil microbial biomass, and microbial community composition in grafted and non-grafted star anise plantations in Guangxi, China were studied using high-throughput sequencing technology. The results showed that the microbial biomass carbon and phosphorus contents in the soils of grafted star anise were significantly lower and the phosphatase activity was significantly higher than in the soils of non-grafted star anise. In comparison with the soils of non-grafted star anise plantations, the proportions of Proteobacteria, Acidobacteria, Actinobacteria, and WPS-2 decreased and the proportions of Chloroflexi, Planctomycetes, and Verrucomicrobia increased in the grafted star anise plantations. Meanwhile, Bacteroidetes was a dominant bacterial phylum unique to the soil of the grafted star anise plantations. Moreover, the proportions of Ascomycota and Basidiomycota increased and the proportions of Mortierellomycota and unclassified_k_Fungi decreased in the soils of the grafted star anise plantations. Furthermore, Basidiomycota and Rozellomycota had significant dominance in the grafted star anise plantations. In general, grafting can improve soil fertility and maintain soil health by promoting soil nutrient cycling and increasing the soil’s microbial diversity.

## 1. Introduction

Star anise (*Illicium verum* Hook.f.), a broad-leaved evergreen tree, is better known as Chinese star anise and belongs to the Illicium family. Its fruit is used as a spice in Chinese cuisine [1,2]. It is also a highly regarded medicinal plant, and several biologically important phytochemicals have been related to its various medicinal properties such as antibacterial, antiviral, and antioxidant activities [3,4]. Guangxi Zhuang Autonomous Region is not only the origin but also the main producing area of star anise in China and is reputed to be the hometown of star anise. However, in recent years, the extensive management of most of the star anise plantations in Guangxi has led to fluctuations in its yield and quality, which seriously hinders the sustainable development of the star anise industry in Guangxi [1]. Therefore, it is essential to build an environmentally friendly and sustainably managed cultivation system for star anise. Among these systems, grafting is a commonly used and effective management measure in star anise-producing areas. 

Grafting is a horticultural technique in which the rootstock and scion are joined together to form a new plant after the successful connection of the vascular tissue and is widely used to increase plants’ horticultural properties [5]. The success rate of grafting depends on the affinity between the rootstock and scion. Generally speaking, the rootstock and scion used are from the same genus, which has more affinity than rootstocks and scions of different genera [6]. Grafting can not only improve the yield and product quality but can also prolong the postharvest time and lifespan [7,8,9]. In addition, grafting enhances plants’ resistance to different biological and abiotic stress conditions, such as pathogens, temperature, salinity, heavy metals, and water stress in the soil and air [7,10,11,12,13,14]. Moreover, grafting enhances the absorption of nutrients from the soil and improves the nutrient and water utilization abilities of plants [15,16,17]. However, most of the previous indicators used pertain to soil chemistry, and there is a lack of biological indicators for soil and its microbial properties. 

Soil’s enzymatic activity is mainly of microbial origin, being derived from intracellular, cell-associated, or free enzymes, which play an important role in maintaining the soil’s fertility and health [18]. Soil microbial biomass serves as a crucial bio-indicator for assessing soil quality [19]. A higher biomass signifies the enhanced capacity of the soil to provide nutrients to plants via the mineralization of organic matter [20]. Specifically, soil microbial biomass carbon (MBC) plays a dual role: it fosters the formation of highly active new humus in the soil and sensitively reflects subtle soil changes even before variations in the total soil carbon content become apparent [21]. Soil microbial biomass nitrogen (MBN) is indicative of the soil’s nitrogen availability and is pivotal to the supply and circulation of nitrogen within the soil [21]. Soil microbial biomass phosphorus (MBP), although not directly absorbable by plants, contributes to the slow release of inorganic phosphorus through its turnover, thus serving as a significant source of available phosphorus, vital to plant growth [22]. Additionally, MBP reflects the supply level of phosphorus in the soil [23], further emphasizing its importance in soil fertility and plant nutrition.

Soil microorganisms, particularly bacteria and fungi, occupy a pivotal position in terrestrial ecosystems, mediating nutrient cycling, material transformation, energy flow, and information transfer [24,25]. Bacteria, the most populous and diverse microbial group in soil [26], contribute significantly to the mineralization of organic debris, the sequestration of humus within the soil mineral layer, and the facilitation of the carbon and nitrogen cycles, ultimately enriching plant growth with essential nutrients [27,28,29,30]. Fungi, primarily known as decomposers in ecosystems [31,32], form beneficial symbiotic associations with the plant roots, enhancing nutrient acquisition (especially nitrogen and phosphorus), stress resistance, pest and disease defense [33,34], and soil structure [35]. Notably, variations in the soil’s microbial activity and community composition serve as sensitive indicators of the soil ecosystem’s health and quality [18], underscoring their importance in maintaining the soil’s fertility and plant productivity.

As the effects of grafting on the soil fertility and health of star anise plantations are still unknown, this limits our understanding of the improvements that grafting mechanisms make to the yield of star anise, i.e., whether grafting leads to greater soil fertility and whether the soil fertility and soil microbial community in star anise plantations are changed by grafting. Therefore, it is of great significance to analyze the soil’s enzyme activity, soil microbial biomass content, and the soil’s microbial diversity characteristics in grafted star anise plantations to clarify the effect of grafting mechanisms on improving soil fertility and maintaining soil health in star anise plantations.

## 2. Materials and Methods

### 2.1. Study Site and Soil Sampling

Soil samples from grafted [self-rooted grafting of the same seeding, the rootstock was an annual seedling (about 0.5 cm in diameter), and an annual bearing branch with excellent characteristics was used as the scion] and non-grafted [the primary star anise without grafting] star anise plantations with identical tree ages (16 years) were collected from the Paiyangshan forestry station (107°5′ E, 22°1′ N), Ningming County, Guangxi, China. The average yearly temperature is 22 ℃ in this area, which is in the subtropical monsoon climatic zone. The soil type of the study area was acid red loam and the basic soil chemical properties of the experimental site, including the pH (5.46), soil organic matter (12.9 g kg^−1^), total nitrogen (0.81 g kg^−1^), total phosphorus (0.39 g kg^−1^), total potassium (2.68 g kg^−1^), alkaline nitrogen (53.7 mg kg^−1^), available phosphorus (9.1 mg kg^−1^), and available potassium (89.0 mg kg^−1^) were determined by referring to our previous methods [36].

In November 2019, the topsoil and impurities were removed first, and then five soil samples from depths of 0~30 cm under the canopy of star anise trees were collected, respectively. Five trees were selected randomly from each plantation; five soil samples were collected and mixed into biological replicates, and three replicates were set for each plantation, respectively. 

Sterile sealing bags were used to collect the soil samples and incubators with ice packs were used to transport them. Then, the soil samples were divided into three parts: one part was stored at −80 °C for soil DNA extraction, another part was stored at 4 °C for enzyme activity and microbial biomass analyses, and the last part was used for the determination of soil chemical properties after being air-dried in a room.

### 2.2. Analysis of Soil Biological Properties

The β-glucosidase, aminopeptidase, and phosphatase activities were determined by Hayano [37], Ladd [38], and Tabatabai and Bremner [39], respectively. The soil microbial biomass carbon, nitrogen, and phosphorus were determined by Vance et al. [40], Joergensen and Brookes [41], and Powlson et al. [19], respectively. 

### 2.3. Analysis of Soil Microbial Diversity

We used the FastDNA^®^ Spin Kit for Soil (MP Biomedicals, Santa Ana, CA, USA) to extract the soil microbial community genomic DNA. The extraction, PCR amplification, and sequencing of the total DNA of the soil samples were performed following previously described protocols [1,36]. The hypervariable region V3-V4 of the bacterial 16S rRNA gene was amplified with the primer pairs 338F and 806R, and the region ITS1 of the fungal ITS gene was amplified with the primer pairs ITS1F and ITS2R [1,36]. We deposited the raw reads of both bacteria and fungi in the NCBI Sequence Read Archive (SRA) database (Accession Number: PRJNA987439). The data processing and microbial diversity analysis were the same as those in our previous study [1,24,36]. 

### 2.4. Statistical Analysis

The mean value was compared using an independent *t*-test in SPSS 26 with a significance level of 0.05. The results were presented as the mean and standard deviation (mean ± SD). We used Excel 2019 and IBM SPSS Statistics 21 to analyze the experimental data and used the Majorbio Cloud Platform (www.majorbio.com, accessed on 1 March 2023) to conduct the online microbial data analysis [1,36]. 

## 3. Results

### 3.1. Soil Enzyme Activities

The differences in the soil enzyme activities between the grafted and non-grafted star anise plantations are shown in Table 1. No significant difference was found between the plantations in terms of the activity of β-glucosidase (*p* > 0.05). However, the activities of phosphatase and aminopeptidase all significantly changed (*p* < 0.05). Among them, the activities of phosphatase and aminopeptidase in the grafted star anise plantation were significantly higher and lower, respectively, than those in the non-grafted plantation (*p* < 0.05). This indicated that the soil phosphorus cycling process in the grafted star anise plantation was more active than that in the non-grafted plantation. However, the nitrogen cycling process in the grafted plantation was less active than that of the non-grafted plantation.

### 3.2. Soil Microbial Biomass

The soil microbial biomass in the grafted and non-grafted star anise plantations is shown in Table 2. Except for soil microbial N, which did not differ significantly between the plantations (*p* > 0.05), the soil microbial biomass C and P in the grafted star anise plantation were significantly lower than in the non-grafted plantation (*p* < 0.05). This indicated that the soil nutrient pool in the grafted plantation was lower than that of the non-grafted plantation.

### 3.3. Soil Microbiological Diversity and Community Analysis

**Figure 1:**
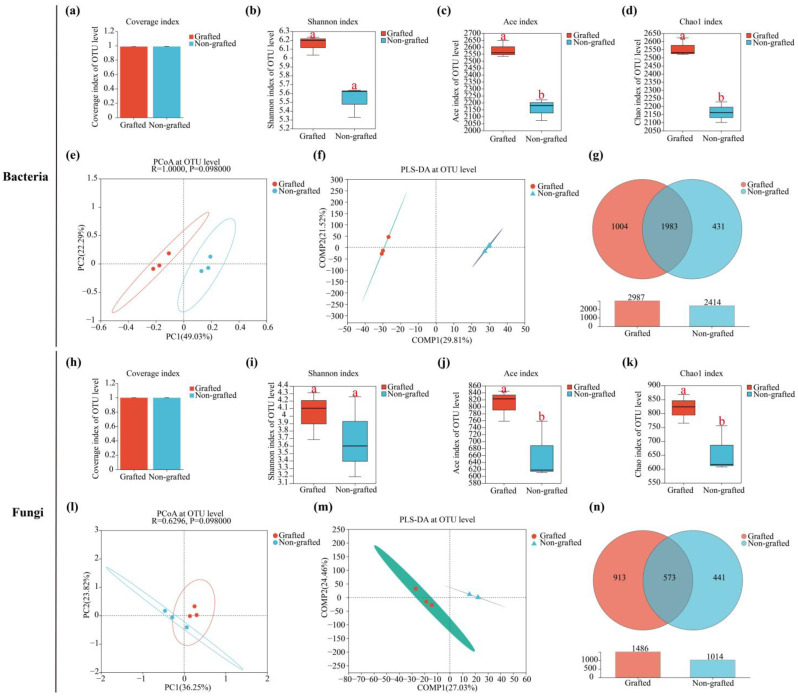
Soil bacterial and fungal Alpha and Beta diversities between grafted and non-grafted star arise plantations at the OTU level. (**a**) Bacterial coverage index. (**b**) Bacterial Shannon index. (**c**) Bacterial Ace index. (**d**) Bacterial Chao1 index. (**e**) Bacterial PCoA plot. (**f**) Bacterial PLS-DA plot. (**g**) Bacterial Venn diagram. (**h**) Fungal coverage index. (**i**) Fungal Shannon index. (**j**) Fungal Ace index. (**k**) Fungal Chao1 index. (**l**) Fungal PCoA plot. (**m**) Fungal PLS-DA plot. (**n**) Fungal Venn diagram. Different lower-case letters indicate significant differences between treatments at *p* < 0.05.

The sequencing data were reliable, as the soil bacterial and fungal coverage rates reached 99.00% (Figure 1a) and 100% (Figure 1h), respectively. In comparison with the non-grafted star anise, the soil bacterial Shannon index in the grafted star anise plantation was not significant (*p* > 0.05) (Figure 1b), and the bacterial Ace and Chao1 indices were all significantly different (*p* < 0.05) (Figure 1c,d). Similarly, the differences in the soil bacterial Shannon, Ace, and Chao1 indices between the grafted and non-grafted star anise plantations were also found for soil fungi (Figure 1i–k). The Shannon index was used to describe microbial (i.e., bacterial and fungal in this study) diversity, and the Ace and Chao1 indices were used to describe microbial richness. These results indicated that the soil microbial diversity and richness in the grafted star anise plantation were higher than for the non-grafted plantation, and there was a significant difference in the microbial richness (*p* < 0.05).

Principal Co-ordinate Analysis (PCoA, Bray–Curtis, ANOSIM) was performed at the OTU level to evaluate the extent of the similarities in the soil bacterial and fungal communities between the grafted and non-grafted star anise plantations, respectively (Figure 1e,l). Meanwhile, Partial Least Squares Discriminant Analysis (PLS-DA) was also carried out to evaluate the differences in the soil bacterial and fungal communities between the grafted and non-grafted star anise plantations, respectively (Figure 1f,m). These results showed that the soil bacterial communities clustered separately in each of the plantations, indicating that the soil bacterial communities differed, though not significantly (*p* > 0.05).

The diversity estimates, at the OTU level, of the soil bacterial and fungal communities are shown as Venn diagrams in Figure 1g and Figure 1n, respectively. There were 1004 and 431 unique soil-dominant bacterial OTUs in the grafted and non-grafted star anise plantations, respectively (Figure 1g), and 913 and 441 unique soil-dominant fungal OTUs between the grafted and non-grafted star anise plantations, respectively (Figure 1n). These results showed that the numbers of unique soil bacterial and fungal OTUs were increased through grafting. 

**Figure 2:**
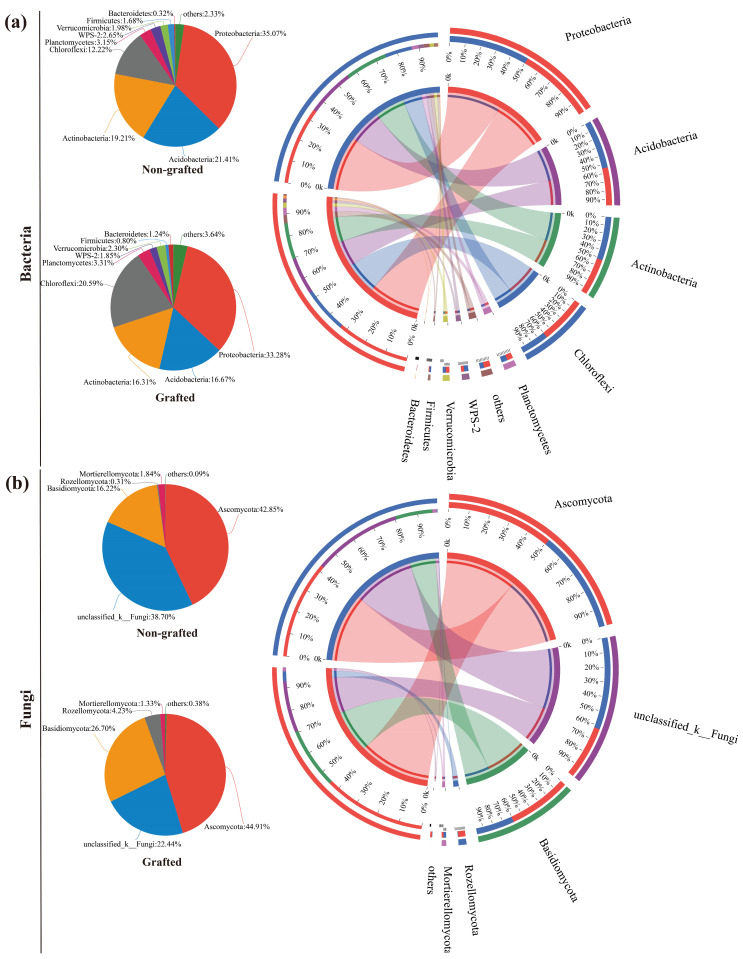
Soil bacterial (**a**) and fungal (**b**) compositions of grafted and non-grafted star arise plantations at the phylum level.

As seen in Figure 2a, all eight soil-dominant (relative abundances greater than 1%) bacteria phyla were detected in the grafted and non-grafted star anise plantations at the phylum level. In the grafted star anise plantation, Proteobacteria (33.28%), Acidobacteria (16.67%), Actinobacteria (16.31%), Chloroflexi (20.59%), Planctomycetes (3.31%), WPS-2 (1.85%), Verrucomicrobia (2.30%), Bacteroidetes (1.24%), and others (3.63%) were the soil-dominant bacterial phyla. In contrast, Proteobacteria (35.07%), Acidobacteria (21.41%), Actinobacteria (19.21%), Chloroflexi (12.22%), Planctomycetes (3.15%), WPS-2 (2.65%), Verrucomicrobia (1.98%), Firmicutes (1.68%), and others (2.33%) were the soil-dominant bacterial phyla in the non-grafted star anise plantation. All of the above results suggested that not only had the proportions of soil-dominant bacterial phyla changed, but the soil bacterial compositions, such as those of Firmicutes and Bacteroidetes, were also altered by grafting (Figure 2a). On the other hand, five and four soil-dominant fungal phyla were found in the grafted and non-grafted star anise plantations, respectively (Figure 2b). Ascomycota (44.91%), Basidiomycota (26.70%), unclassified_k_Fungi (22.44%), Rozellomycota (4.23%), and Mortierellomycota (1.33%) were the most dominant fungal phyla in the soil of the grafted star arise plantation. In contrast, Ascomycota (42.85%), unclassified_k_Fungi (38.70%), Basidiomycota (16.22%), and Mortierellomycota (1.84%) were the most dominant fungal phyla in the soil of the non-grafted star anise plantation. These results suggested that not only did the proportions of the soil fungal phyla change, but the soil fungal compositions in the star anise plantations were also altered through grafting (Figure 2b).

**Figure 3:**
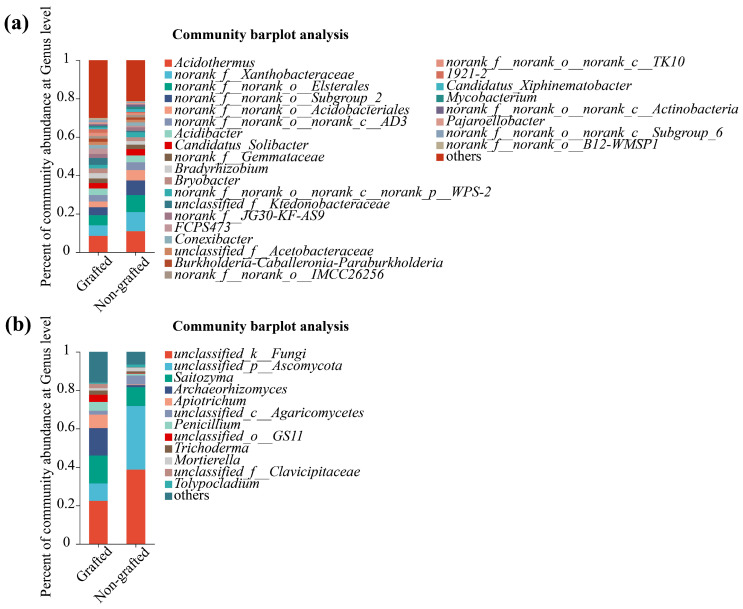
Soil bacterial (**a**) and fungal (**b**) compositions of grafted and non-grafted star anise plantations at genus level.

As shown in Figure 3a, the numbers of soil-dominant bacterial genera between the grafted and non-grafted star anise plantations were 24 and 20, respectively. Among them, *unclassified_f__Ktedonobacteraceae*, *FCPS473*, *norank_f__norank_o__norank_c__TK10*, *1921-2*, *Pajaroellobacter*, *norank_f__norank_o__norank_c__Subgroup_6*, and *norank_f__norank_o__B12-WMSP1* were the unique soil-dominant bacterial genera in the grafted star anise plantations. In contrast, *Candidatus_Xiphinematobacter*, *Mycobacterium*, and *norank_f__norank_o__norank_c__Actinobacteria* were the unique soil-dominant bacterial genera in the non-grafted star anise plantation (Figure 3a). As shown in Figure 3b, the numbers of soil-dominant fungi between the grafted and non-grafted star anise plantations were 11 and 7, respectively. Among them, *Apiotrichum*, *Penicillium*, *unclassified_o_GS11*, *Trichoderma*, and *unclassified_f_Clavicipitaceae* were the unique soil-dominant fungal genera in the grafted star anise plantations, and *Tolypocladium* was the unique soil-dominant fungal genus in the non-grafted star anise plantation (Figure 3b).

### 3.4. Soil Microbiological LEfSe Analysis and Function Prediction

**Figure 4:**
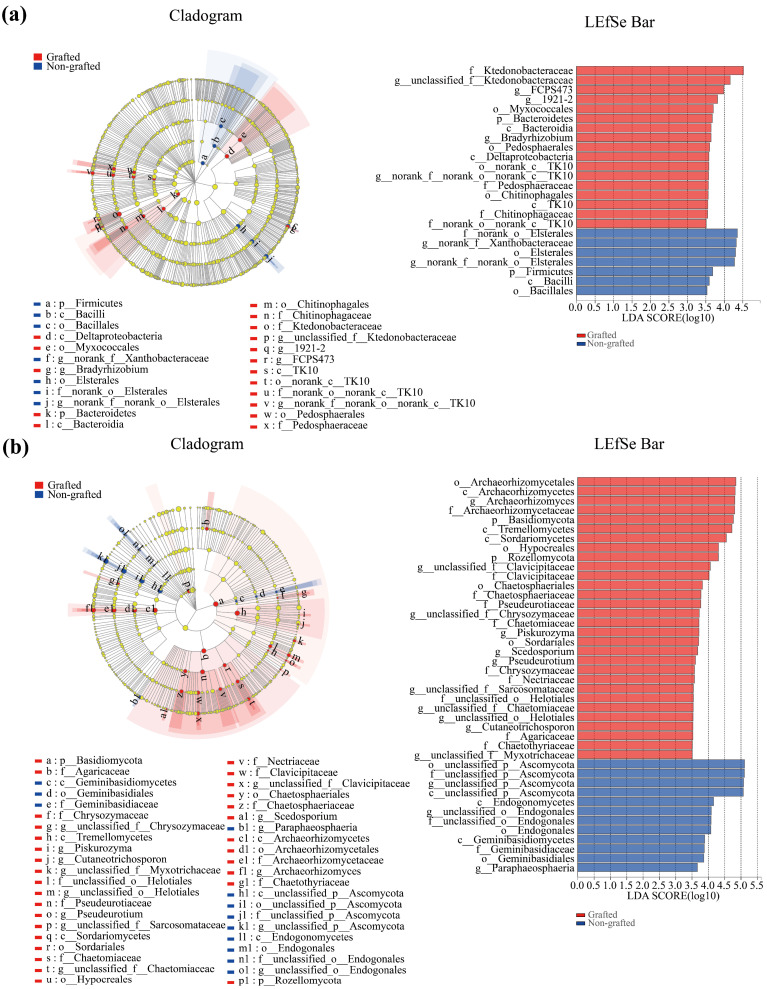
LEfSe analysis of soil bacteria (**a**) and fungi (**b**) in grafted and non-grafted star anise plantations (LAD score = 3.5).

An evolutionary branching diagram was created showing all of the hierarchical relationships, from phylum to genus, of each taxonomic unit from the inner to the outer circles. At the phylum level, Bacteroidetes and Firmicutes were the most dominant in the grafted and non-grafted star anise plantations, respectively. At the genus level, *unclassified_f_Ktedonobacteraceae*, *FCPS473*, *1921-2*, *Bradyrhizobium*, and *norank_f_norank_o_norank_c_TK10* exhibited significant dominance in the grafted star anise plantation. In contrast, *norank_f_Xanthobacteraceae* and *norank_f_norank_o_Elsterales* were the most dominant in the non-grafted star anise plantation (Figure 4a). In addition, at the phylum level, Basidiomycota and Rozellomycota exhibited significant dominance in the grafted star anise plantations. At the genus level, *Archaeorhizomyces*, *unclassified_f_Clavicipitaceae*, *unclassified_f_Chrysozymaceae*, *Piskurozyma*, *Scedosporium*, *Pseudeurotium*, *unclassified_f_*_*Sarcosomataceae*, *unclassified_f_*_*Chaetomiaceae*, *unclassified_o__Helotiales*, *Cutaneotrichosporon*, and *unclassified_f__Myxotrichaceae* were the most dominant in the grafted star anise plantation, while *unclassified_p__Ascomycota*, *unclassified_o__Endogonales*, and *Paraphaeosphaeria* were the most dominant in the non-grafted star anise plantation (Figure 4b).

**Figure 5:**
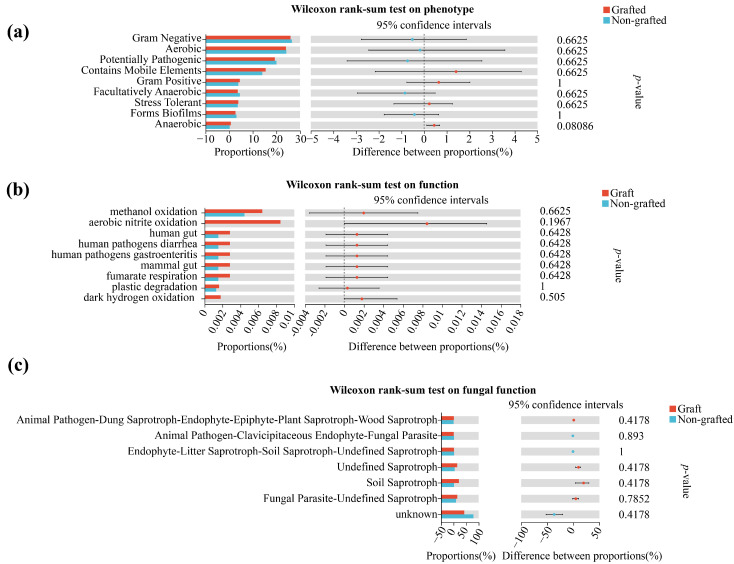
Function predictions of the soil bacteria (**a**,**b**) and fungi (**c**) in the grafted and non-grafted star anise plantations.

The soil bacterial phenotypes were mainly classified into nine groups using BugBase analysis and a Wilcoxon rank-sum test was performed for the grafted and non-grafted star anise plantations (Figure 5a). The results showed that the abundance of these nine bacterial phenotypes did not differ between the plantations. Moreover, the abundant percentages for the phenotypes contain Mobile Elements and Stress Tolerant were higher in the grafted star anise plantation than in the non-grafted plantation. The soil bacterial and fungal functions were predicted using FAPROTAX (Figure 5b) and FUNGuild (Figure 5c), respectively. Meanwhile, the functions of the bacterial and fungal communities in each plantation were evaluated using a Wilcoxon rank-sum test (*p* < 0.05). The results showed that the functions of the soil bacteria (Figure 5b) and fungi (Figure 5c) in the grafted star anise plantation did not differ significantly from those in the non-grafted star anise plantation.

### 3.5. Correlation Network Analysis

**Figure 6:**
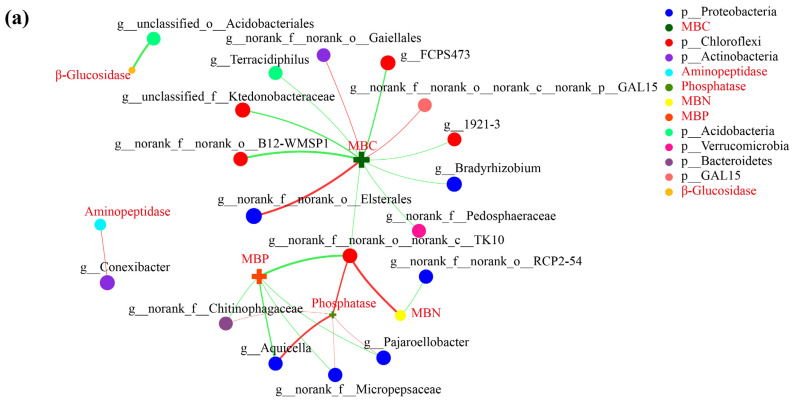

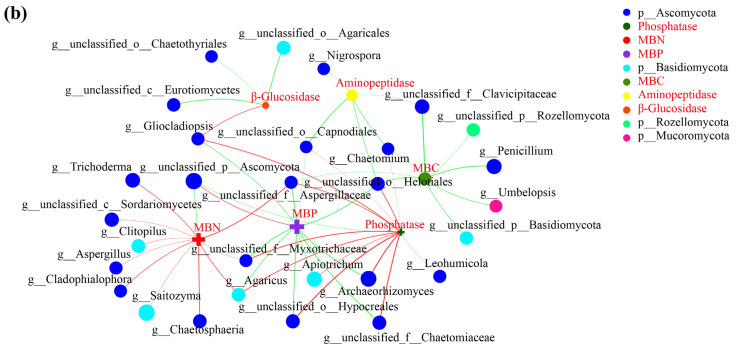
Two-factor correlation network analysis of the top 50 bacterial (**a**) and fungal (**b**) genera and soil environmental factors at the genus level. The Spearman correlation coefficients were computed to elucidate the relationship between soil environmental factors and microbial genera. A significant correlation was considered when the absolute value of the correlation coefficient was ≥0.5, with a *p*-value < 0.05. These data were visualized using a graph where the node size denoted species abundance, and distinct colors were assigned to different species. Furthermore, connecting lines were colored to represent the nature of the correlation, with red and green signifying positive and negative correlations, respectively. The thickness of these lines was proportional to the magnitude of the correlation coefficient, with thicker lines representing stronger correlations. The density of the lines provided an insight into the closeness of the connection between the nodes. The prefixes “g” and “p” indicated genus and phylum, respectively.

In order to further understand the influence of soil environmental factors (soil enzyme activity and microbial biomass) on soil microorganisms in the grafted and non-grafted star anise plantations, the top 50 bacterial (Figure 6a) and fungal (Figure 6b) genera were selected to construct a two-factor correlation network analysis with soil enzyme activities (β-glucosidase, phosphatase, and aminopeptidase) and soil microbial biomass contents (MBC, MBN, and MBP), respectively. The results showed that phosphatase, MBC, and MBP were correlated with more bacterial genera, while phosphatase, MBN, and MBP were correlated with more fungal genera. We also found that soil environmental factors were more likely to be associated with more fungal genera than soil bacteria, indicating that soil fungi were more likely to be affected by soil environmental factors in the star anise plantations.

## 4. Discussion

### 4.1. Response of Soil Enzyme Activity and Microbial Biomass to Grafting in Star Anise Plantations

Ling et al. [42] reported that the β-glucosidase activity (the function of decomposing labile carbon) in the soil of watermelon did not significantly change after grafting, and the activity of *N*-acetylglucosaminidase (the function of hydrolyzing nitrogen) was significantly lower in the soil of grafted watermelon than in that of non-grafted watermelon. We also found similar results, and we used aminopeptidase activity to describe the function of hydrolyzing nitrogen during the N cycle process in the present study. In another study, the phosphatase activity of cucumber rhizosphere soil was significantly increased through grafting, and the soil microbial biomass C in the grafted cucumber was significantly lower than in the non-grafted cucumber [43]. The same results were found in our study. Wang et al. [44] reported that the rhizosphere soil phosphatase activity and the soil microbial biomass C of *Xanthoceras sorbifolia* was significantly increased after grafting. The change in microbial biomass C was inconsistent with our results, which may be due to the different kinds of plant species under study.

### 4.2. Response of Soil Microbial Diversity to Grafting in Star Anise Plantations

Ogundeji et al. [45] found that the soil bacterial and fungal diversity and richness of eggplant did not significantly change after grafting alone or combined with other cultivation strategies, such as bio-fumigation and biochar application (*p* > 0.05), although the soil bacterial diversity and richness increased to varying degrees. We found similar results, but the difference was that the soil bacterial and fungal richness significantly increased in our study (Figure 1). 

In addition, we found that the number and quantity of unique and total soil-dominant bacterial and fungal OTUs increased after grafting, which was consistent with the results of Ogundeji et al. [45].

This result was attributed to the benefit of grafting rootstocks to soil microbial growth [13]. Previous studies have shown that grafting stimulates microbial growth by affecting the composition of root secretions [5,46], which is thought to provide a carbon source and energy source for microorganisms [47]. This explains the higher microbial diversity in the grafted plants than in the non-grafted plants [48].

### 4.3. Response of Soil Microbial Community to Grafting in Star Anise Plantations

Previous studies confirmed that soil microorganisms closely interact with plants [49]. In particular, rhizospheric soil microorganisms have been shown to have important effects on plant productivity and agro-ecosystem function [50]. Meanwhile, soil properties and plant species also affect soil microbial community structures and their functions [51]. For example, the change observed in the plant community was not only related to variations in the soil bacterial community structure but the microbial community was also synchronized with the visible change in the dominant plants [52]. It was reported that soil microbial community composition and activity are significantly affected by plant genotypes [53]. For example, plant genotypes contributed to the shaping of the dynamic bacterial communities associated with the roots of rice plants [54]. Marasco et al. [55] reported that soil environmental factors and plant genotypes could regulate the recruitment and selection of plant-related microbiota. Our results also found that the soil microbial diversity and community structure were quite different between the grafted and non-grafted star anise plantations. The differences in the root secretion content and quantity between grafted and non-grafted plants [56] may alter soil microbial compositions.

In the present study, Acidobacteria was a top phylum in both plantations, which is in agreement with the findings in [45]. As potential core taxa, the dominant and differentially abundant microbial taxa played vital ecological roles in microbiome assembly and ecosystem functioning [57,58]. It has been reported that Chloroflexi can decompose cellulose, starch, and long-chain sugars and use nitrate for energy to become involved in C cycling and nitrification processes and tends to grow in a nutrient-rich environment [45,59,60]; we also found that the proportion of dominant Chloroflexi increased in the grafted star anise plantation to a greater extent than in the non-grafted plantation. Moreover, Bacteroidetes are the primary degraders of complex carbohydrate-based biomass: they can secrete diverse arrays of carbohydrate-active enzymes (CAZymes), thus promoting plant growth and enhancing biodiversity [61]. Our study also confirmed that Bacteroidetes was one of the most dominant bacteria in the grafted star anise plantation, which was significantly enriched by its presence.

Kuramae et al. [62] found that most fungi species from the Basidiomycota and Ascomycota phyla could degrade cellulose. Our results showed that the relative abundances of Basidiomycota and Ascomycota in the grafted star anise soils were higher than those in the non-grafted soils, which led to resource competition among some microbial communities during the process of decomposition [45]. Previous research confirmed that *Penicillium* was ubiquitous in soil and was considered a key fungal group in phosphorus cycling [63]. The high activity of soil phosphatase promoted the phosphorus cycle in the grafted star anise plantation (Table 1), which may be related to the unique enrichment of *Penicillium*. *Trichoderma* is common in soils, displaying antifungal properties as well as promoting growth and inducing plant resistance against pathogenic fungi [64]. 

In the present study, we found that the C and N that could be directly absorbed and utilized by the plants in the grafted star anise plantations were more abundant than in the non-grafted plantations. In addition, the soil fertility of the grafted star anise was lower than that of the non-grafted star anise, and the microbial diversity and richness in the grafted star anise soil were higher than in the soil of the non-grafted star anise. Studies have shown that plant roots can release different types of organic matter (exudates, secretions, sloughed-off cells) into the soil and provide a relatively stable environment for microbial growth [65].

## 5. Conclusions

In star anise plantations, we found that grafting promotes soil nutrient circulation by increasing soil enzyme activities, decreasing microbial biomass C and P, increasing the soil microbial community diversity, and enriching unique favorable microorganisms. 

## Figures and Tables

**Table 1:** Soil enzyme activities between grafted and non-grafted star anise plantations (n mol g^−1^ min^−1^, 30 °C).

Samples	β-Glucosidase	Aminopeptidase	Phosphatase
Grafted	0.27 ± 0.03 a	15.40 ± 0.87 b	0.23 ± 0.03 a
Non-grafted	0.26 ± 0.03 a	16.67 ± 0.53 a	0.08 ± 0.01 b

Note: Different lowercase letters in the same column represent significant differences (*p* < 0.05).

**Table 2:** Soil microbial biomass between grafted and non-grafted star anise plantations (mg kg^−1^).

Samples	Microbial Biomass C	Microbial Biomass N	Microbial Biomass P
Grafted	121.86 ± 7.61 b	7.17 ± 0.77 a	25.90 ± 1.44 b
Non-grafted	144.64 ± 7.34 a	6.55 ± 0.39 a	52.40 ± 4.37 a

Note: Different lowercase letters in the same column represent significant differences (*p* < 0.05).

## Data Availability

The raw data for soil bacterial and fungal sequencing were deposited in the NCBI Sequence Read Archive (SRA) database under accession number PRJNA987439.
